# Properties of Transmission and Leaky Modes in a Plasmonic Waveguide Constructed by Periodic Subwavelength Metallic Hollow Blocks

**DOI:** 10.1038/srep14461

**Published:** 2015-09-25

**Authors:** Jin Jei Wu, Chien Jang Wu, Jian Qi Shen, Da Jun Hou, Wen Chen Lo

**Affiliations:** 1 Department of Electrical Engineering, Chung Hua University, Hsinchu 300, Taiwan; 2 Institute of Electro-Optical Science and Technology, National Taiwan Normal University, Taipei 116, Taiwan; 3 Centre for Optical and Electromagnetic Research, State Key Laboratory of Modern Optical Instrumentations, Zhejiang University, Hangzhou 310058, China

## Abstract

Based on the concept of low-frequency spoof surface plasmon polaritons (spoof SPPs), a kind of leaky mode is proposed in a waveguide made of a subwavelength metal-block array with open slots. Numerical results reveal that a new transmission mode is found in the periodic subwavelength metal open blocks. This modal field is located inside the interior of a hollow block compared with that in a solid metal block array. The dispersion curve shows that such a new SPPs mode has a negative slope, crossing the light line, and then going into a zone of leaky mode at higher frequencies. The leaky mode has a wider frequency bandwidth, and this can lead to a radiation scanning angle of 53° together with high radiation efficiency. Based on the individual characteristics exhibited by a frequency-dependent radiation pattern for the present leaky mode, the waveguide structure can have potential applications such as frequency dividers and demultiplexers. Experimental verification of such a leaky mode at microwave has been performed, and the experimental results are found to be consistent with the theoretical analysis.

Metamaterials, which are fabricated by artificial metallic structures, possess novel electromagnetic (EM) properties and have attracted much attention in the community. In 2004, by introducing periodic subwavelength slots, Pendry *et al.* proposed a kind of new structure to mimic the transmission property of surface plasmon polaritons (SPPs) at low frequencies^1^,^2^. SPPs are caused by the strong coupling between the EM field and the free electrons inside a metal with a negative permittivity in visible region^3^,^4^, and they are highly confined near the metal surface. Such SPPs can possibly be applied in photonic circuits in order to increase the integration density of optical devices and to suppress the interference between adjacent waveguides. Several kinds of SPP-based waveguides have been put forward for reducing the size of waveguides^5^,^6^. It is highly expected that such a strongly localized mode can also be obtained not only in the visible region but also at low frequencies such as terahertz (THz) and millimeter as well. Since free electrons in the metal cannot be efficiently coupled to the EM field at low frequencies, the SPPs mode is not easy to be guided around a flat metal surface. To resolve these problems, Pendry *et al.* proposed a structure of periodic subwavelength holes on a metal surface in order that the EM field can be dramatically trapped. Such a trapping mechanism was later explained in detail in ref. 7. The geometry-controlled SPPs, which are referred to as the spoof SPPs (SSPPs), were first experimentally verified at microwave by taking advantage of subwavelength rectangular metal holes^8^. A periodic subwavelength corrugated metal surface with an EM field confined tightly on it has been widely investigated for THz applications. For instance, in order to transmit THz signals, a novel kind of cylindrical metal wires with transmission properties controlled by geometric parameters of lattice constant was proposed^9^,^10^. A new technique for highly efficient conversion of surface-plasmon-like modes to spatial radiated modes has been suggested based on spoof surface plasmon polariton emitters^11^.

As far as the new waveguide structure supporting spoof SPPs is concerned, a one-dimensional (1D) periodic Domino plasmonic waveguide was theoretically analyzed in THz regime in ref. 12 and then experimentally verified in ref. 13. Another kind of subwavelength periodic V-shaped channel waveguide with low bending loss was also reported^14^. These THz subwavelength periodic structures can be more flexible than before in design of new metallic waveguide devices. Besides, a kind of microwave subwavelength periodically corrugated metal wires covered by dielectric layer with high dielectric constant was studied^15^, and 1D Domino array was also investigated at microwave^16^,^17^.

In the literature, most studies, in which the properties of SSPPs in a periodic Domino structure have been widely theoretically and experimentally investigated, focused on the analysis of field distribution and dispersion characteristics^16^,^17^. In addition, accurate *S*-parameter properties are also essential for a waveguide device. For example, a new kind of SSPPs based on metallic UV channel waveguide (with a structure of alternating arrangement of U- and V-shaped grooves) in microwave regime was proposed in ref. 18, where the authors measured the S-parameters by vector network analyzer. For a 1D Domino array structure, the transmission properties can be measured through a waveguide converter technique^19^,^20^. A possible application of such SSPPs is that they can provide a low crosstalk transmission line in microwave integrated circuits^21^,^22^,^23^. For example, a microstrip line, which has a structure of corrugated gradient grooves, can serve as a potential device of broadband molecular sensing^24^. Other interesting applications include ultra-thin SSPPs with CPW feed structures^25^,^26^ and SSPPs-assisted deep-subwavelength negative-index waveguiding technique^27^.

Less attention has been paid to the properties of leaky modes in aforementioned works. Very recently, Cui *et al.* have suggested a new scheme for controllably manipulating electromagnetic radiation by holographic metasurfaces (a structure of subwavelength metallic patches on grounded dielectric substrates), where some interesting topics relevant to leaky waves have been investigated both theoretically and experimentally^28^,^29^. The hybrid metasurface that is composed of planar metamaterial and holographic metasurface has also been developed aiming at simultaneous controls of propagating waves and surface waves (including leaky-surface waves)^30^. Now in this work, we shall propose a new kind of leaky modes radiation based on the concept of low-frequency SSPPs in a periodic subwavelength structure. By tuning the geometric parameters, the corresponding dispersion can be obtained and therefore the desired transmission and radiation properties can be achieved. The dispersion curves will be calculated by making use of the commercial software, COMSOL. We shall consider a THz metallic waveguide constructed by periodic subwavelength metallic blocks with open slots. It will be found that an additional SPPs mode exists in such periodic block array. The corresponding dispersion curve lies within the light cone and thus corresponds to a leaky mode. Compared with a transmission mode, the propagation constant of the leaky mode has a complex form, *k*_*z*_ = *β* + *jα*. This means there is radiation loss under the assumption that the metal is identified with a perfect electric conductor (PEC). An experiment will be carried out to confirm the theoretical results.

## Results

### Transmission properties of a THz plasmonic waveguide

**Figure 1:**
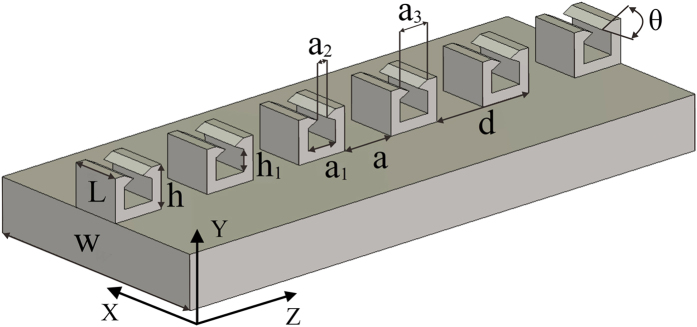
Waveguide based on a periodic subwavelength metallic block array supporting spoof SPPs, in which the width is *L*, the period is *d*, the adjacent spacing is *a*, the height is *h*, and the waveguide lateral width is *w*. In a single subwavelength metallic hollow block, the bottom length is *a*_1_, the mouth length is *a*_2_, the aperture height is *h*_1_, and the opening angle is *θ*.

The proposed plasmonic waveguide made of a periodic subwavelength metallic bevel hollow block array structure, of which the geometric parameters are shown in Fig. 1. For simplicity, we assume the metal to be a PEC in our simulation. This is a reasonable approximation at microwave and THz^1^,^2^. The plasmonic waveguide with a periodic metallic block array will be simulated by the finite element method (FEM), from which the dispersion curve, the field distribution and the transmission coefficient can be obtained.

**Figure 2:**
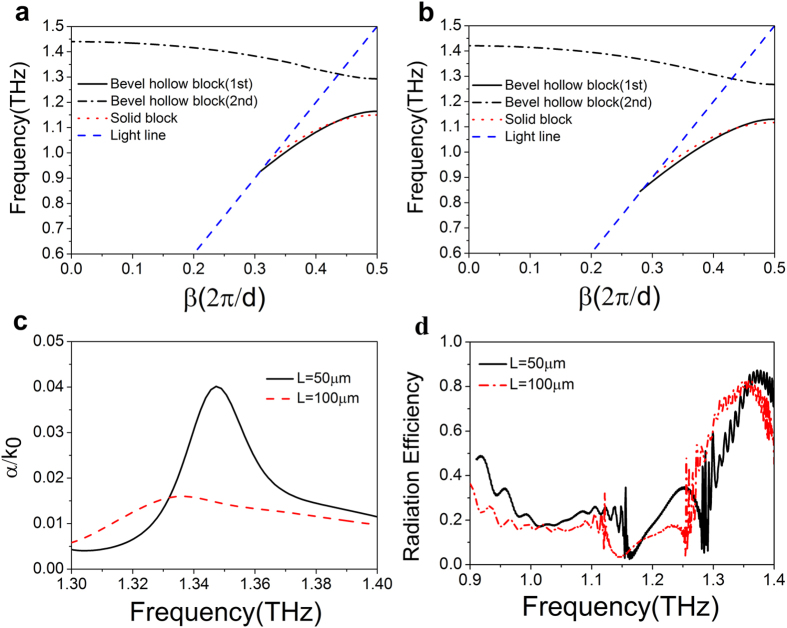
Dispersion curves of plasmonic waveguides. (**a**) Dispersion curves of the periodic groove plasmonic waveguide for the case with dimensions *d* = 100 μm, *a* = 50 μm, *L* = 50 μm, *h* = 40 μm and *θ* =  53.4°. The solid black curve and the dash-dotted curve represent the first and the second SPPs modes, respectively, of the metallic hollow block, and the dotted curve represents the SPPs mode of the metallic solid block. (**b**) Dispersion of the periodic groove plasmonic waveguide with *L* = 100 μm. (**c**) Attenuation constant of the leaky modes of the two periodic groove plasmonic waveguides with *L* = 50 μm and *L* = 100 μm. (**d**) Leakage radiation efficiency as a function of frequency for *L* = 50 μm and *L* = 100 μm.

The dispersion curves (*f* vs. *β*) of the plasmonic waveguide are displayed in Fig. 2(a). Here, *β* is restricted in the first Brillouin zone of 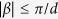. For the periodic metallic hollow block array, the geometric parameters are given by *d* = 100 μm, *a* = 0.5*d*, *L* = 50 μm, *h* = 40 μm, *a*_2_ = 10 μm, *a*_*3*_ = 30 μm, *h*_1_ = 20 μm, and *θ* = 53.4°. We shall focus on the fundamental mode and the leaky mode of this plasmonic waveguide. The fundamental mode (as a guided mode) locates on the right side of the light line. The dispersion curve of the solid block array is also given for the purpose of comparison. For the period metallic solid block array (SBA) with *d* = 100 μm, *a* = 0.5*d*, *L* = 50 μm, and *h* = 40 μm, the cutoff frequency *f*_*sc*_ is 0.9338 THz, the asymptotic frequency *f*_*ss*_ (*β* = *π*/*d*) is 1.124 THz, and the working bandwidth is 0.1902 THz, as can be seen in the red dotted curve. For the period metallic hollow block array, the cutoff frequency *f*_1*c*_ of the fundamental mode is 0.928 THz, the asymptotic frequency *f*_1*s*_ (*β* = *π*/*d*) is 1.1645 THz, and the working bandwidth is 0.237 THz, as shown in the black solid curve. With the same geometric parameters, the hollow block array and the solid block array both have a similar asymptotic frequency, but the hollow block array has a wider transmission band due to smaller cutoff frequency. Except for the above-mentioned guided mode, there exists a new leaky mode for the hollow block array in Fig. 2(a) with asymptotic frequency *f*_2*s*_ = 1.2927 THz. This new dispersion curve intersects with the light line at the frequency *f* = 1.312 THz, and becomes a leaky mode in the radiation zone until 1.44 THz with the leaky mode bandwidth of 0.128 THz. Figure 2(b) shows the first and the second modes of the hollow block plasmonic waveguide with a larger waveguide width (*L* = 100 μm). The fundamental mode has cutoff frequency of 0.84498 THz, asymptotic frequency of 1.13029 THz, and transmission bandwidth of 0.28531 THz. Compared with the structure with *L* = 50 μm, both cutoff frequency and asymptotic frequency of the structure of *L* = 100 μm decrease. Whereas, the transmission bandwidth of *L* = 100 μm increases. The second mode has asymptotic frequency of 1.2676 THz, and the dispersion line passes through the light line at 1.2905 THz and becomes a leaky mode until 1.42062 THz. The propagation constant of the leaky mode has a complex form of *k*_*Z*_ = *β* + *jα*, in which the imaginary part represents radiation loss from the block array. Figure 2(c) shows the imaginary part of propagation constant *α* for the periodic hollow block array. Here, the imaginary parts for the two cases (*L* = 50 μm and *L* = 100 μm) are shown in solid and dashed curves, respectively. For *L* = 50 μm, the imaginary part, *α*/*k*_0_ (with *k*_0_ = 2*π*/*λ*_0_ the wave number in free space), increases in the leaky mode band from *α*/*k*_0_ = 0.00422 at the frequency of *f* = 1.3 THz and has a peak value of *α*/*k*_0_ = 0.04 at *f* = 1.3476 THz and then drops to 0.01127 at *f* = 1.4 THz. This result suggests that such a plasmonic waveguide can be used as a highly efficient THz antenna. For *L* = 100μm, the imaginary part, *α*/*k*_0_, increases from 0.00577 at *f* = 1.3 THz to the maximum of 0.0162 at *f* = 1.33748 THz, and then drops to 0.00966 at *f* = 1.4 THz. The peak value of *α*/*k*_0_ becomes smaller when the block cell is wider. This means the radiation efficiency of the THz antenna can be controllable by the width of the block cell. In Fig. 2(d) are the leakage radiation efficiency (defined as 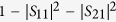 for the two waveguides). The result in Fig. 2(c) is also confirmed in Fig. 2(d), i.e., the radiation efficiency decreases significantly in the leakage regime when the metallic block width changes from *L* = 50 μm to *L* = 100 μm. In the guiding regime, however, the electromagnetic field is confined more tightly in the periodic structure of metallic blocks of *L* = 100 μm than that of *L* = 50 μm.

**Figure 3:**
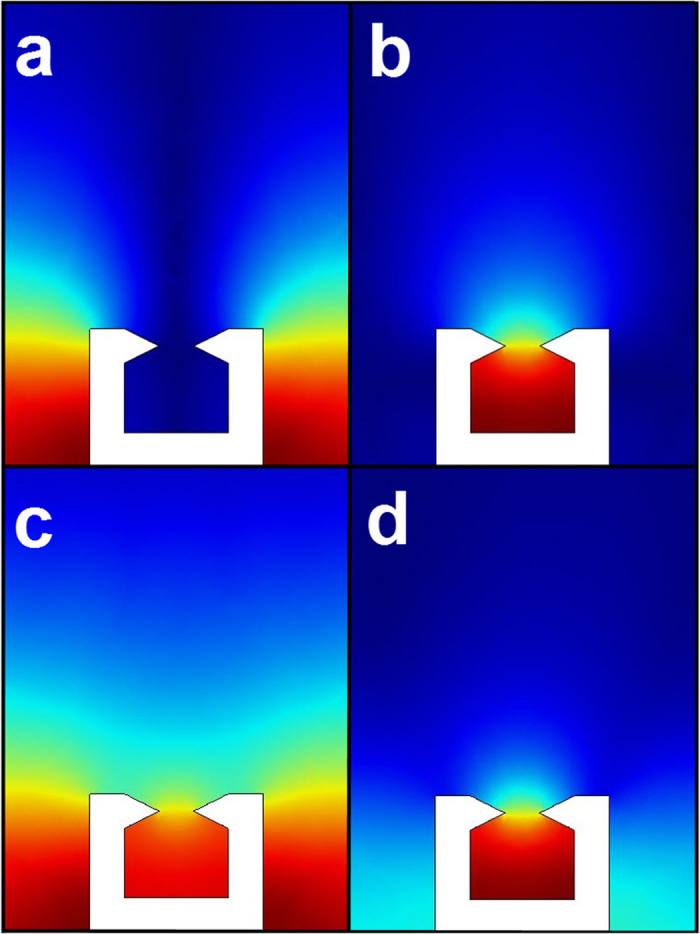
Magnetic field distribution for a periodic metallic hollow block array at (**a**) *β* = *π*/*d* (*f* = 1.1645 THz), at (**b**) *β* = *π*/*d* (*f* = 1.2927 THz), at (**c**) *β* = 0.7*π*/*d* (*f* = 1.00256 THz), and at (**d**) *β* = 0.7*π*/*d* (*f* = 1.3606 THz).

The magnetic field distribution in a unit cell at four typical frequencies is presented in Fig. 3. In Fig. 3(a) we show the field profile of the fundamental mode at the asymptotic frequency of *f*_1s_ = 1.1645 THz (*β* = *π*/*d*) for *L* = 50 μm. It can be seen that most of the EM field spreads out of the block, much similar to the case in solid block, implying that these two structures have almost the same asymptotic frequency. In Fig. 3(b), we have distribution of the second mode at the asymptotic frequency of *f*_2s_ = 1.2927 THz (*β* = *π*/*d*), in which most of the EM field is localized inside the block. In addition, the EM field is uniformly distributed both inside and outside the block at *f* = 1.00256 THz (*β* = 0.7*π*/*d*) for the fundamental mode, while, for the second mode, most of the EM field is distributed inside the block at *f* = 1.3606 THz (*β* = 0.7*π*/*d*).

**Figure 4:**
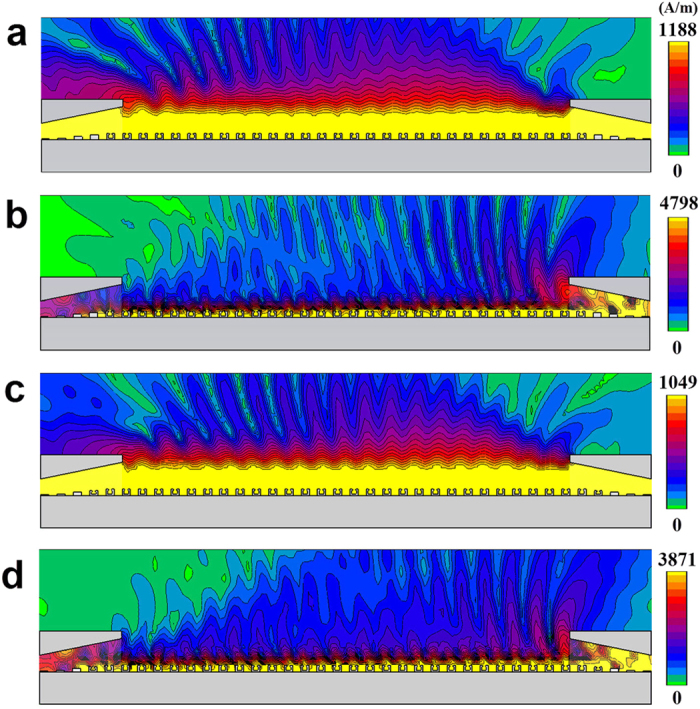
Simulated magnetic near-field distribution of SPPs plasmonic waveguides (periodic metallic hollow block waveguides). The widths of the unit cells are taken to be *L* = 50 μm and 100 μm, respectively. (**a**) The magnetic-field distribution at *β* = 0.7*π*/*d* (*f* = 1.00256 THz) and *L* = 50 μm for the periodic metallic hollow block waveguide. (**b**) The magnetic-field distribution at *β* = 0.7*π*/*d* (*f* = 1.3606 THz) and *L* = 50 μm. (**c**) The magnetic field distribution at *β* = 0.7*π*/*d* (*f* = 0.97417 THz) and *L* = 100 μm. (**d**) The magnetic field distribution at *β* = 0.7*π*/*d* (*f* = 1.33531 THz) and *L* = 100 μm.

In Fig. 4, we plot the magnetic near-field distributions of the plasmonic waveguides at *β* = 0.7*π*/*d*, for *L* = 50 μm and 100 μm, respectively. In Fig. 4(a) is the magnetic near-field distribution of the fundamental mode at *f* = 1.00256 THz for *L* = 50 μm. It can be clearly seen that the EM field is highly confined near the surface of the plasmonic waveguide. In Fig. 4(b), we show the near-field distribution of the leaky mode at *f* = 1.3606 THz for *L* = 50 μm. The EM field is input at the right end and it can be radiated from the waveguide into the free space at a specific angle. Fig. 4(c) is the magnetic near-field distribution at *f* = 0.97417 THz for *L* = 100 μm, which is similar to the field profile for *L* = 50 μm at *f* = 1.00256 THz. However, the structure with *L* = 100 μm has exhibited an effect of strong field confinement. Fig. 4(d) is the magnetic near-field distribution of the leaky mode at *f* = 1.33531 THz for *L* = 100 μm.

**Figure 5:**
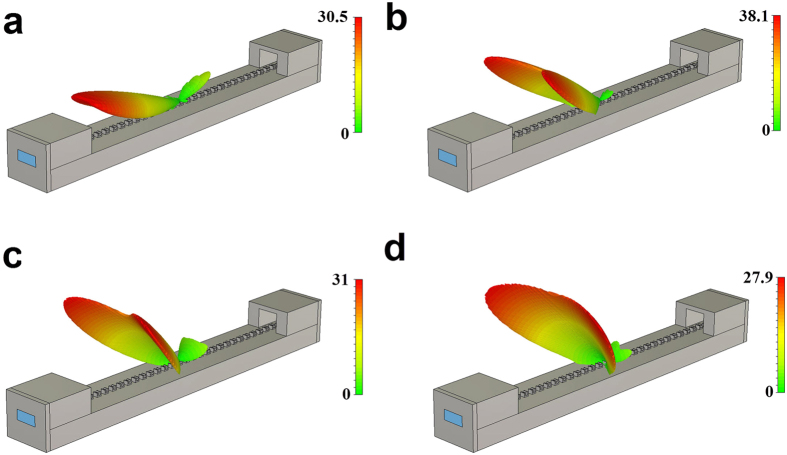
Simulated far-field distribution of the spoof SPPs plasmonic waveguide with hollow block (unit cell) width *L* = 50 μm at four different frequencies: *f* = 1.3058 (**a**), 1.3326 (**b**), 1.3538 (**c**), and 1.3708 THz (**d**).

Shown in Fig. 5(a–d) is the far-field distribution of the leaky mode at frequencies 1.3058 THz, 1.3326 THz, 1.3538 THz and 1.3708 THz, respectively. The corresponding radiation angles are *ϕ* = 286°, *ϕ* = 303°, *ϕ* = 312° and *ϕ* = 320°, respectively, for *L* = 50 μm. Since the present plasmonic waveguide is a subwavelength periodic structure, there must be a number of space harmonics with phase constant *β*_*n*_. When 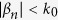, the electromagnetic field will be radiated from the metallic periodic structure to the free space. It is evident that the radiation direction, which can be evaluated by 
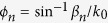
 in ref. 31, scans as the frequency changes.

In the above analysis, the metal is assumed to be PEC, so that there is no loss for the transmission mode. In the realistic case, however, there exists a metallic loss due to the surface current in metal with a finite conductivity. We will study the metallic loss using an aluminum waveguide. The lattice constant of our periodic waveguide is *d* = 100 μm, and so the waveguide can work in the THz band. The metallic loss can be calculated with the perturbation method^22^, from which we have 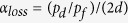, where *p*_*d*_ is the total metallic loss in a periodic unit and *p*_*f*_ is the total transmission power. The propagation length is thus given by 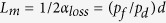.

**Figure 6:**
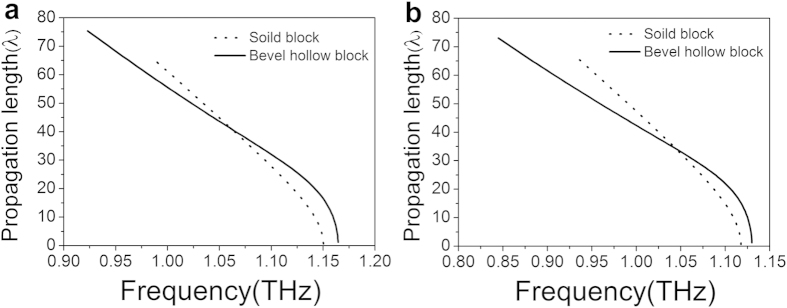
Normalized propagation length for (**a**) the plasmonic waveguide with *L* = 50 μm and (**b**) the plasmonic waveguide with *L* = 100 μm.

The normalized propagation length (*L*_*m*_/λ) as a function of frequency for the fundamental mode is shown in Fig. 6, where the normalized propagation length of the solid block array is also plotted in the dotted line for the purpose of comparison. At low frequencies, the propagation length is large for the solid block array compared with the hollow block array, because there is a significant metallic loss for the latter, in which more EM field is confined near the metal surface. The transmission lengths of these two cases decrease as the frequency increases and become zero at the asymptotic frequency. In addition, at a certain frequency (e.g., at 1.067 THz), both curves (for propagation length in hollow and solid block array) have an intersection for *L* = 50 μm.

## Experimental verification of plasmonic waveguide in microwave band

**Figure 7:**
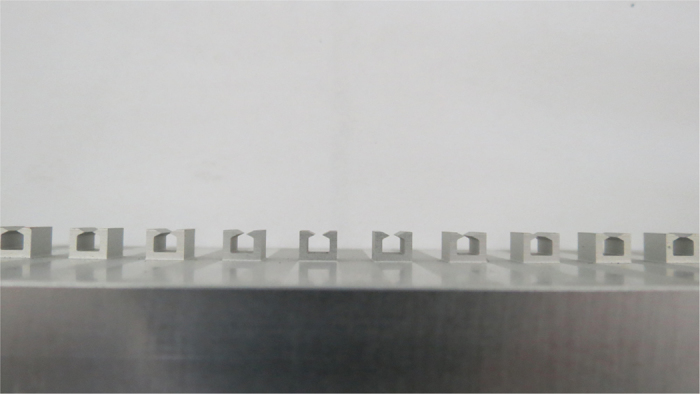
Picture of the experimental plasmonic waveguide.

Since it is difficult to fabricate a waveguide at THz, as an alternative by scaling down the frequency, we design and fabricate a metallic structure in Fig. 7 with aluminum to verify the characteristics of a plasmonic waveguide in the microwave regime. At each end of the waveguide, there is a transition region of 50 mm in length and the height of the periodic metallic hollow block array is gradually increased. As a result, the field distribution from the rectangular waveguide is gradually transformed into the spoof SPPs field distribution along the transition region, reducing the reflection in the junction between the plasmonic waveguide and the feeding waveguide. Here, *h* = 4 mm, *d* = 10 mm, *a* = 0.5*d*, *L* = 5 mm, *w* = 50 mm, and the length of the plasmonic waveguide *t* = 375 mm. Some other geometric parameters are *h*_1_ = 2 mm, *a*_1_ = 3 mm, and *a*_2_ = 1 mm.

**Figure 8:**
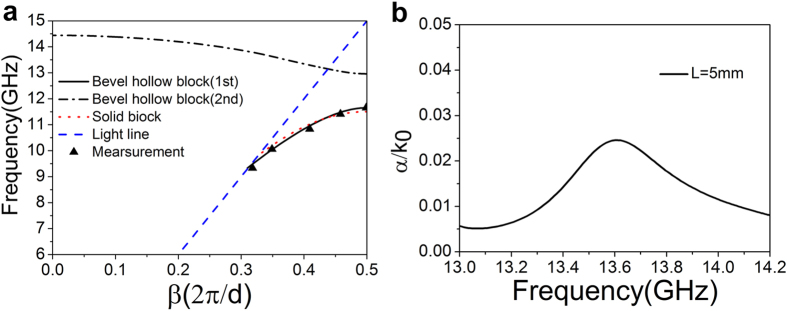
Dispersion curves and propagation constant. (**a**) Dispersion curves of the plasmonic waveguide with the geometric parameters: *h* = 4.0 mm, *d* = 10 mm, *a* = 0.5*d*, *L* = 5 mm, and *θ* = 53.4°. (**b**) The imaginary part of the propagation constant as a function of frequency.

The simulated dispersion curves at microwave are given in Fig. 8(a). It can be found that there are two modes for such a plasmonic waveguide. The results agree with those at THz. For the fundamental mode plotted in the solid black curve, there is a cutoff frequency at *f*_3*c*_ = 9.3328 GHz and an asymptotic frequency at *f*_3*s*_ = 11.6524 GHz. Thus, the transmission bandwidth is 2.3195 GHz. For the comparison purpose, the guided mode of solid block array is plotted in the dotted curve from which we find a cutoff frequency, *f*_4c_ = 9.873 GHz, an asymptotic frequency *f*_4s_ = 11.503 GHz, and consequently the transmission bandwidth of 1.63041 GHz, smaller than that of the hollow block array. The measured dispersion curve of such a mode is indicated by the triangle marks in Fig. 8(a). For the second mode of the hollow block array in the dashed curve, the asymptotic frequency is *f*_5s_ = 12.959 GHz, and the dispersion curve intersects with light line at *f* = 13.15 GHz and then enters into the leaky mode zone until *f* = 14.443 GHz. The propagation constant is complex, i.e., *k*_*z*_ = *β* + *jα* for a leaky mode. In Fig. 8(b), we show the imaginary part *α*/*k*_0_ as a function of frequency. It is found that *α*/*k*_0_ = 0.00659 at 13.2 GHz, 0.02459 at 13.611 GHz, and afterwards drops to 0.00798 at *f* = 14.2 GHz. In addition, this structure has a maximum leakage radiation efficiency at *f* = 13.611 GHz.

**Figure 9:**
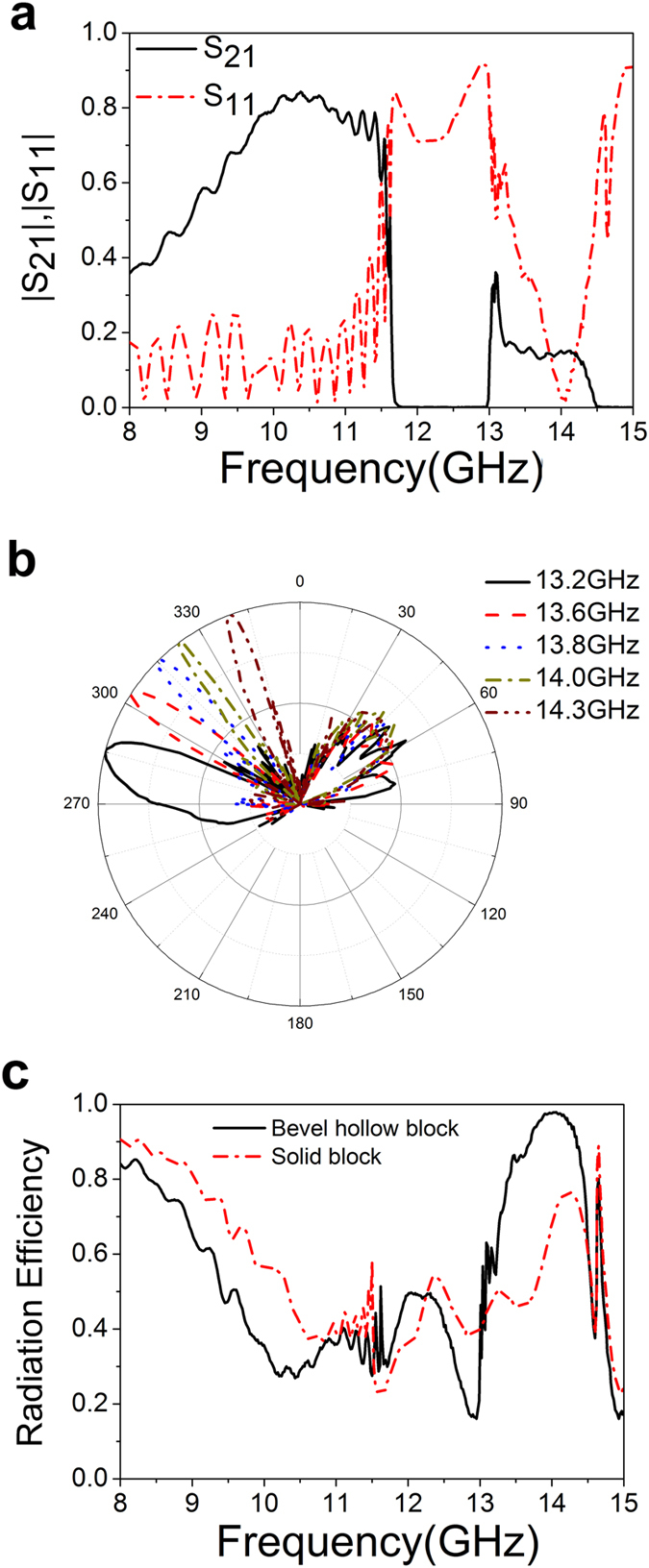
Experimental results of the plasmonic waveguide. (**a**) Measured S parameters of the plasmonic waveguide with geometric parameters of *h* = 4.0 mm, 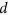 = 10 mm, *a* = 0.5*d*, *L* = 5 mm, and *θ* = 53.4°. (**b**) Far field radiation pattern of the plasmonic waveguide. (**c**) Leakage radiation efficiency as a function of frequency.

To study the transmission properties, an aluminum waveguide with a length of 375 mm is fabricated and measured by Vector Network Analyzer (VNA). Fig. 9(a) displays the S parameters for the case with geometric parameters: *h* = 4 mm, *d* = 10 mm, *a* = 0.5*d*, *L* = 5 mm and *w* = 50 mm. It can be seen that *S*_21_ is 0.358 at 8 GHz, and then it increases up to the maximum value of 0.843 at 10.38 GHz, decreases to 0.507 at 11.62 GHz, and drops sharply to 0.068 at 11.66 GHz. On the other hand, *S*_11_ is 0.1774 at 8 GHz, and is relatively large in the band gap between 11.66 GHz and 12.96 GHz. In the leakage zone between 13.11 GHz and 14.5 GHz, S_11_ is smaller than 0.5 while *S*_21_ is smaller than 0.2, and hence 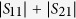 has a much smaller value 0.155 at the frequency of 14.02 GHz. Hence, the experimental results for the main characteristics of guided mode and leaky mode agree well with the theoretical simulation. Fig. 9(b) presents the far field radiation pattern for *L*  = 5.0 mm. At 13.2 GHz, the radiation direction of main beam is 285°, and the half power beam width (HPBW) is Δ*ϕ* = 16°. At the frequency 13.8 GHz, the radiation angle is 315°, and the HPBW is Δ*ϕ* = 5°, and at 14.3 GHz, the radiation angle is 339° and the HPBW is Δ*ϕ* = 5°. In Fig. 9(c) is the leakage radiation efficiency for the two waveguides. The confinement effect in the solid block array is also plotted in the dotted curve for comparison. Apparently it is a guided mode between 8 GHz and 11.5 GHz, in which the leakage radiation efficiency of bevel hollow block waveguide is smaller than that of the solid block waveguide. This is due to the fact that the EM field is highly confined for the additional hollow block in that region. From 13.15 GHz to 14.443 GHz, the bevel hollow block waveguide enters into the leaky mode zone, where the leakage radiation efficiency is much higher than that in the solid block waveguide, agreeing with the theoretical results. It is noteworthy that the leakage radiation efficiency is 97.8% at 14 GHz.

From the previous analysis, the plasmonic waveguide with these geometric parameters has an effect of high confinement of the modal field. As a result, the EM field from the input can be efficiently guided to the output by such a metallic hollow block array. On the other hand, such a hollow block array has an additional leaky radiation mode, which gives rise to frequency scanning radiation. The slight difference between the experimental results and the theoretical simulation is caused by the fabrication precision and the metallic loss.

## Discussion

In conclusion, we have numerically and experimentally studied the properties of transmission and leaky modes in a plasmonic waveguide constructed by periodic subwavelength metallic hollow blocks. The structure can support spoof SPPs whose modal field is highly confined to the structure surface. It is emphasized that, in addition to the transmission mode, there is an extra leaky mode induced by the metallic open apertures, and hence the leakage mode can lead to the effect of frequency scanning radiation. Our analysis indicates that the transmission bandwidth and leaky radiation efficiency can be controllably manipulated via SSPPs waveguide geometric parameter adjustment. Such a kind of waveguide structures can be employed in high power THz, microwave transmission or high directive radiation systems.

## Method

The dispersion curves, radiation efficiency and the ohmic losses of the periodic waveguide structures were calculated by commercial FEM software (COMSOL). The near field distribution and far field radiation pattern of the waveguides were simulated by CST Microwave Studio. The aluminum waveguides at microwave frequencies were fabricated by a computer numerical control (CNC) machine. The performance of the fabricated waveguides such as transmission bandwidth, leaky radiation efficiency and far field radiation pattern were tested through experiments.

## Additional Information

**How to cite this article**: Jei Wu, J. *et al.* Properties of Transmission and Leaky Modes in a Plasmonic Waveguide constructed by Periodic Subwavelength Metallic Hollow Blocks. *Sci. Rep.* **5**, 14461; doi: 10.1038/srep14461 (2015).
